# Pelvic Renal Ectopia: Unusual Position Abnormality

**DOI:** 10.7759/cureus.14365

**Published:** 2021-04-08

**Authors:** Jose C Alvarez, Alexander Joaquin Guerra Mieles, Carlos Rivera-Escalante, Amilkar Rodriguez-Arrieta, Luis Antonio Rodriguez Arrieta

**Affiliations:** 1 Internal Medicine, University of Antioquia, Medellin, COL; 2 Radiology and Diagnostic Images, Hospital Rosario Pumarejo De López, Valledupar, COL; 3 Faculty of Medicine, University of Magdalena, Santa Marta, COL; 4 Faculty of Medicine, University of Cartagena, Cartagena, COL; 5 Endocrinology, University of Antioquia, Medellín, COL

**Keywords:** ectopic kidney, renal ptosis, congenital anomalies of kidney

## Abstract

Pelvic renal ectopia is a congenital anomaly secondary to poor renal migration to the lower back. Usually, these pathologies are of asymptomatic course, therefore its finding is usually fortuitous during radiological examinations for other causes or in work-up of the infrequent symptomatic cases characterized by the occurrence of recurrent infections or symptoms of obstructive uropathy. The objective of this report is to present a case of a 37-year-old female with the unusual manifestation of left pelvic renal ectopia. She was intervened for acute lithiasic cholecystitis, and radiologic techniques diagnosed left pelvic renal ectopia. An updated review of the literature is performed. Despite the anomaly, the patient's renal function tests were normal, so only cholecystectomy was performed without complications.

## Introduction

Congenital renal anomalies consist of wide variability and complexity; these abnormalities are classified according to position and migration (including simple renal ectopia) [1,2]; quantity (either number or volume); differentiation, form, and fusion (including horseshoe kidney and crossed renal ectopia with and without fusion) [3-5]. Renal ectopia may be classified according to its location: pelvic, iliac, abdominal, and thoracic. Pelvic renal ectopia is the most common one, with one out of 3000 cases, with no gender or age preference, and is usually located on the left side [6]. Even though it is usually asymptomatic, abdominal pain, pyuria, and hematuria may be present between the age of 30 and 50 years due to pyelonephritis, abnormalities in the collecting system, lithiasis, or hydronephrosis - the last one being the main complication of a pelvic kidney as it tends to be hypoplastic [7].

## Case presentation

A 37-year-old female patient, multiparous (five pregnancies with five normal labors), with history of a previous episode of abdominal burning pain in right hypochondrium lasting three days, with a 6/10 intensity, associated with alimentary and bilious vomit; an abdominal ultrasound was performed, where thickened gallbladder walls, multiple gallstones, and absence of left kidney was observed. However, she was discharged against medical advice and lost to follow up.

**Figure 1:**
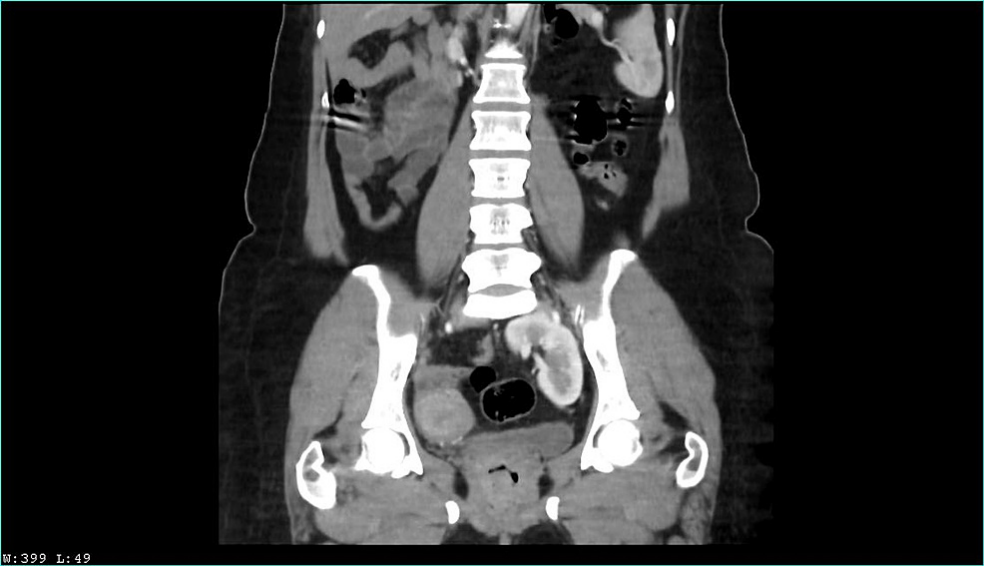
Venous contrast computerized axial tomography scan-Coronal section. Left kidney in the pelvic cavity, located near the left sigmoid, in contact with the upper border of the bladder; no dilation was observed, although the uréter was extremely short. Uptake is homogeneous. Right kidney and collecting system of normal location and anatomy, with no supernumerary kidney.

**Figure 2:**
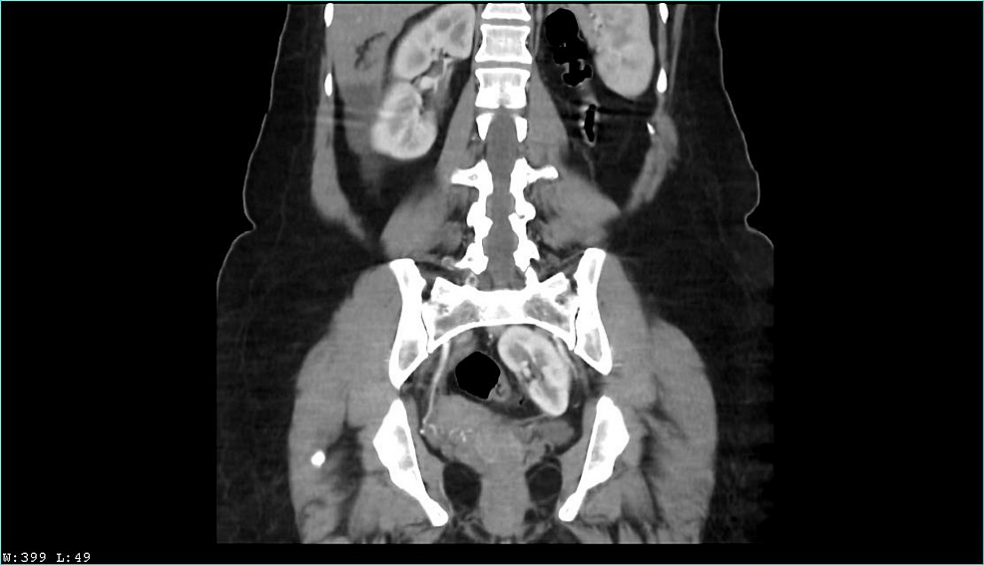
Venous contrast computerized axial tomography scan. Coronal section

**Figure 3:**
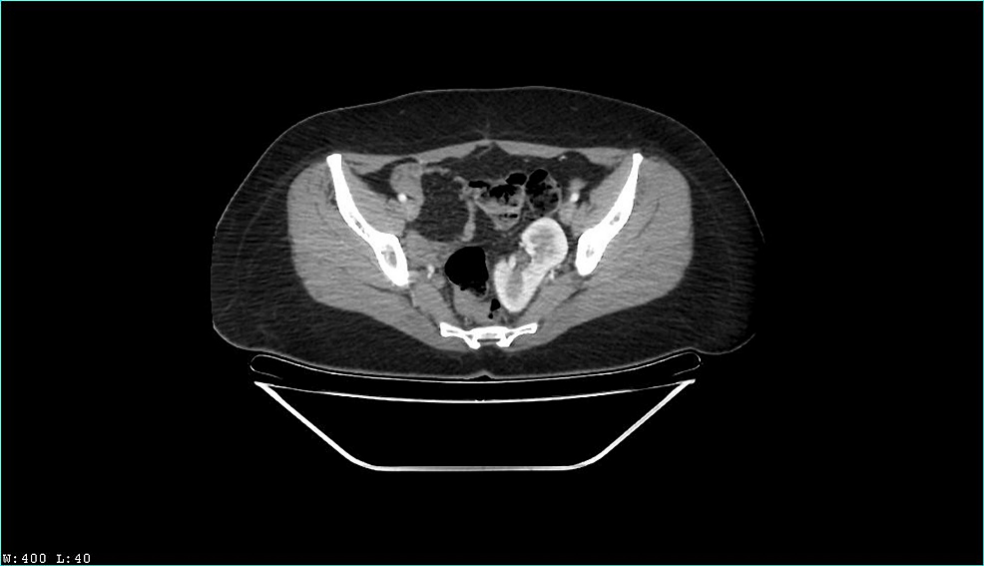
Venous contrast computerized axial tomography scan. Axial section.

Two years later, she presented again with a similar chief complaint; the physical examination revealed a positive Murphy sign in right hypochondrium and painful percussion on the right flank. Abdominal ultrasound revealed as an incidental finding a left renal ectopia abnormally located in the pelvic cavity. A computed tomography urography (CTU) was performed, revealing pelvic nephroptosis with normal size and anatomy. An ambulatory laparoscopic cholecystectomy was scheduled, and the patient was discharged. Nonetheless, the patient presented once again with abdominal pain. A left nephroptosis was suspected by urology, and a new non contrast CTU suggested an hypotrophic left kidney and a supernumerary kidney in the pelvic cavity; therefore, a contrast CTU was performed, with the findings observed in Figure 1, 2, 3, 4. Kidney function tests were normal, and the cholecystectomy was performed without any complications.

**Figure 4:**
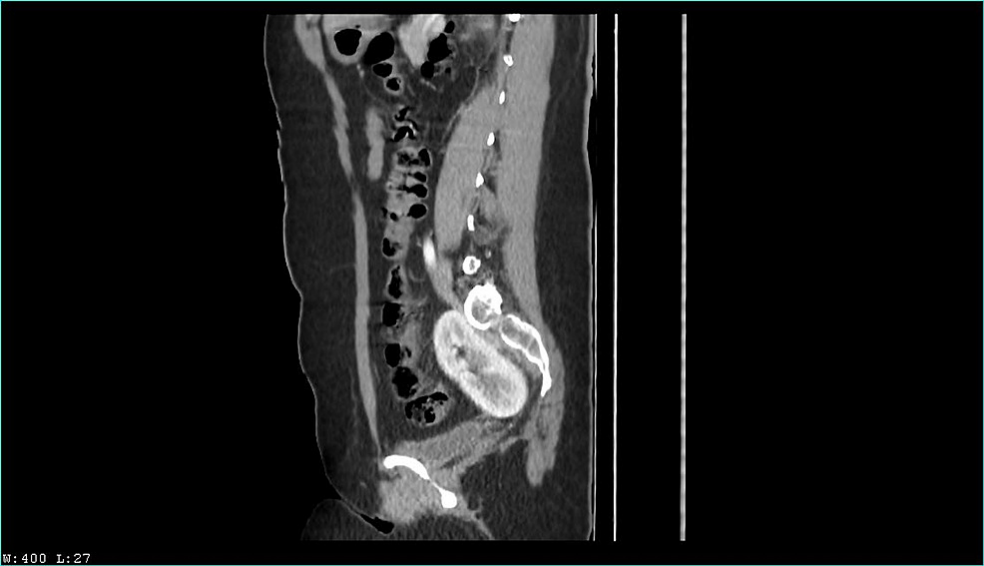
Venous contrast computerized axial tomography scan. Sagital section

## Discussion

Fetal and embryonic development of the kidney and urinary tract is an extremely complex biologic process [8]. The development of a definitive kidney (also known as metanephros) begins on the fifth week of intrauterine life; such development depends on the interaction of two tisular components: the ureteral yolk stemming from the Wolffian duct, which originates the ureters, renal pelvis, calyces and collecting ducts; and the metanephrogenic blastema, undifferentiated mesenchymal cells where nephrons are derived from [3,9].

As kidney development progresses, the ureteral yolk forms the ureter, ascending from its initial pelvic situation to the lumbar position, and it rotates inwards in its longitudinal axis [3]. An ectopic kidney is usually hypoplastic and rotated; the ureter is short and associated with abnormalities, and plenty of small vessels stem from lower parts of the aorta or even the iliac artery [8].

An incidence of 1 out of 50.000 persons has been reported. Most renal ectopia cases remain asymptomatic; confirmed diagnosis is esteemed in only 1 out of 10.000 to 1/30.000 persons [10].

Ectopic kidneys may be asymptomatic, mostly associated with dysplastic abnormalities; in some cases, inadequate drainage may predispose to pyelonephritis, obstruction, and lithiasis [11]. In the adult woman, it may cause labor dystocia; however, in the present case, no dystocia was observed even after five pregnancies or complications due to pelvic renal ectopia [3].

Renal ectopia is different from nephroptosis: the latter is an abnormal location acquired after renal ascension to its natural position. Nephroptosis is due to increased kidney movement in the retroperitoneal space, and is usually observed in obese patients after rapid weight loss; its irrigation is originated in the lumbar aorta, and the ureter has a normal length [12,13]. In the present case, CTU raised concern for nephroptosis and a supernumerary kidney; however, a contrasted CTU confirmed the definitive diagnosis of left renal ectopia of pelvic location - an unusual finding which led to this case report. 

## Conclusions

Pelvic renal ectopia is a congenital anomaly secondary to poor renal migration to the lower back. We present a case of unusual manifestation of left pelvic renal ectopia. Ectopic kidneys may be asymptomatic in the adult woman, it may cause labor dystocia and the main complication of the pelvic kidney as it tends to be hypoplastic for hydronephrosis. The adequate evaluation with diagnostic aids allows us a timely diagnosis and proposes pertinent treatment and surveillance strategies.
